# Anomalous Ferromagnetism of quasiparticle doped holes in cuprate heterostructures revealed using resonant soft X-ray magnetic scattering

**DOI:** 10.1038/s41467-022-31885-1

**Published:** 2022-08-08

**Authors:** B. L. Ong, K. Jayaraman, C. Diao, T. J. Whitcher, A. Jain, H. Hung, M. B. H. Breese, E. S. Tok, A. Rusydi

**Affiliations:** 1 Advanced Research Initiative for Correlated-Electron Systems (ARiCES), Department of Physics, National University of Singapore, 2 Science Drive 3, Singapore, 117551 Singapore; 2 Singapore Synchrotron Light Source, National University of Singapore, 5 Research Link, Singapore, 117603 Singapore; 3 Centre for Advanced 2D Materials, National University of Singapore, 6 Science Drive 2, Singapore, 117546 Singapore; 4 NUS Graduate School for Integrative Sciences and Engineering, Singapore, 117456 Singapore

**Keywords:** Magnetic properties and materials, Electronic properties and materials, Surfaces, interfaces and thin films, Ferromagnetism

## Abstract

We report strong ferromagnetism of quasiparticle doped holes both within the *ab-*plane and along the c-*axis* of Cu-O planes in low-dimensional Au/*d-*La_1.8_Ba_0.2_CuO_4_/LaAlO_3_(001) heterostructures (*d* = 4, 8 and 12 unit-cells) using resonant soft X-ray and magnetic scattering together with X-ray magnetic circular dichroism. Interestingly, ferromagnetism is stronger at a hole doped peak and at an upper Hubbard band of O with spin-polarization degree as high as 40%, revealing strong ferromagnetism of Mottness. For in-*ab*-plane spin-polarizations, the spin of doped holes in O2*p*–Cu3*d*–O2*p* is a triplet state yielding strong ferromagnetism. For out-of-*ab*-plane spin-polarization, while the spins of doped holes in both O2*p*–O2*p* and Cu3*d*–Cu3*d* are triplet states, the spin of doped holes in Cu3*d*–O2*p* is a singlet state yielding ferrimagnetism. A ferromagnetic-(002) Bragg-peak of the doped holes is observed and enhanced as a function of *d* revealing strong ferromagnetism coupling between Cu-O layers along the *c*-axis.

## Abstract (web summary)

Long-range magnetic order of quasiparticle doped holes is important for understanding the physics of cuprate superconductors, albeit difficult to probe in experiments. Ong et al. observe ferromagnetism of quasiparticle doped holes in a cuprate heterostructure and discuss implications for cuprates in the ground state.

## Introduction

One of the unresolved problems in condensed matter physics is elucidating the interplay between long- and short-range interactions of spin of quasiparticle charge carriers, i.e. hole or electron doping, that determines magnetic and electronic structures in strongly correlated electron systems. Of particular interest is copper-oxides (cuprates) of La_2-x_Ba_x_CuO_4_^1^, which is a model system for Mott-Hubbard insulator and high-temperature, unconventional superconductors^2–4^. The cuprates have shown a rich exotic phase diagram in which, as a function of temperature and hole-doping, various exotic phases occur such as antiferromagnetic insulator, pseudogap, spin and charge density waves, spin-glass, strange metal, and superconductivity^5^. It is widely regarded that such a rich and diverse phase diagram is induced by quasiparticle holes doped into the antiferromagnetic insulator. Note that the quasiparticle doped hole is attributed to the extra charge provided by Ba-substitution and is thus not the same as the intrinsic holes from Cu^2+^ provided by the parent compound La_2_CuO_4_. Soon after the discovery of the unconventional superconductivity, ferromagnetism has been theoretically predicted to occur in the hole-doped cuprates^2^. In recent progress, theoretical calculations on one hand suggest that ferromagnetism competes with unconventional superconductivity; while on another hand, other theoretical calculations suggest that ferromagnetism is a precursor or intertwined with the unconventional superconductivity^6–8^. Nevertheless, these theoretical studies have all agreed that ferromagnetism plays an important role in the cuprates and is, therefore, the key to understanding the nature of normal states of the quasiparticle doped holes and their pairing mechanisms responsible for the rich phase diagram in the cuprates. It has been very challenging, however, to directly probe the long-range order of spin of the quasiparticle doped holes, given the overwhelming contribution from the antiferromagnetic ordering of localized Cu spins in the Cu-O plane^8^. Hence, it is a general consensus that this fundamental problem should be experimentally addressed before one can move forward in establishing a reliable model to describe the magnetic and electronic structures of doped holes in Cu-O plane of the cuprates^6,7^. Therefore, an experimental method capable of probing simultaneously long-range magnetic and electronic correlations of the quasiparticle doped holes is critical in addressing this fundamental problem.

## Results and discussions

**Fig. 1:**
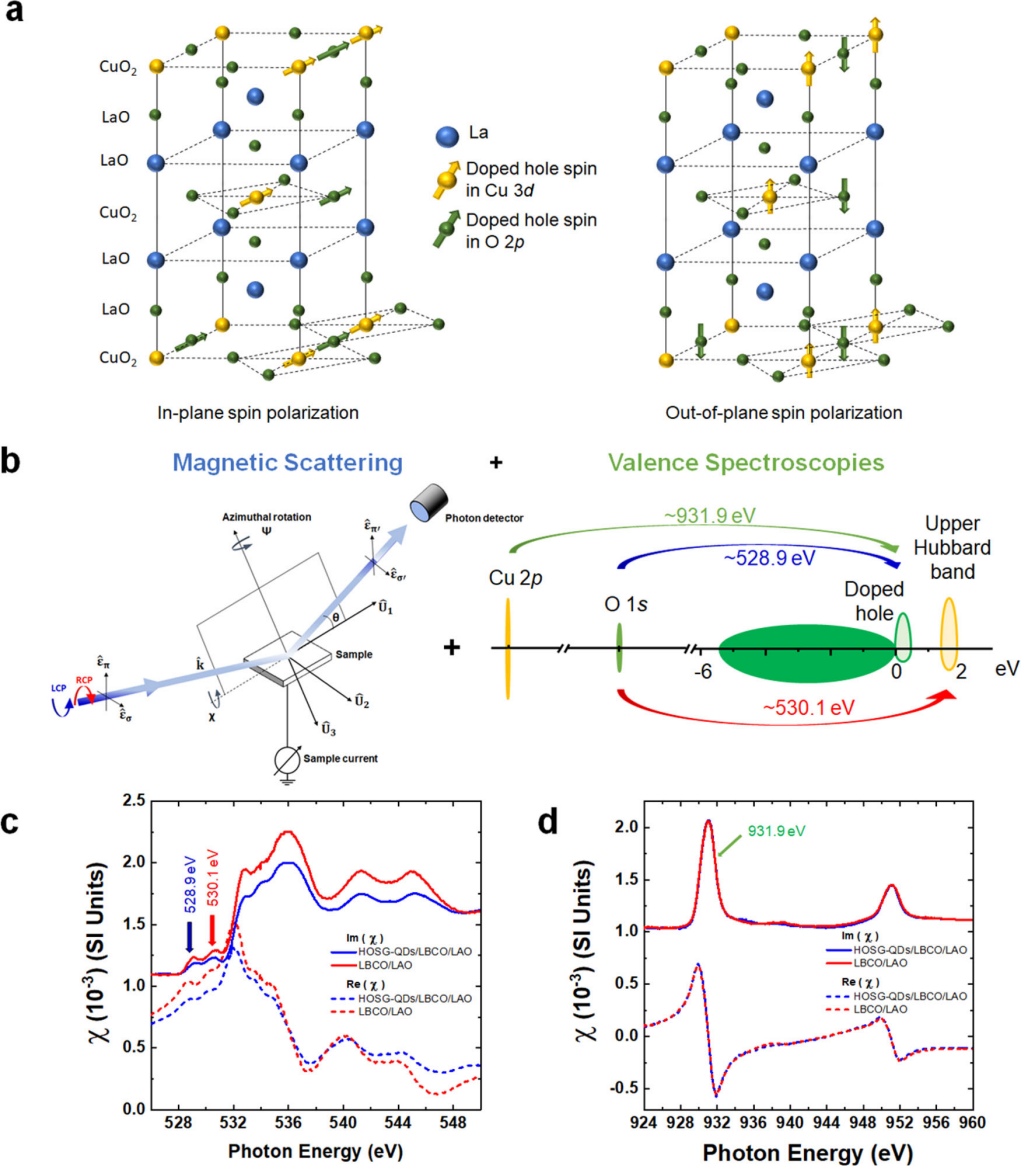
RSXMS, spin structure and complex dielectric susceptibility in HOSG-QDs/La_1.8_Ba_0.2_CuO_4_/LaAlO_3_. **a** The spin structures in HOSG-QDs/La_1.93_Ba_0.07_CuO_4_/LaAlO_3_ showing the in-plane and out-of-plane spin-polarizations. **b** Resonant soft X-ray and magnetic scattering were measured at three different energies showing the scattering geometry and the valence spectroscopies involving these three transition energies. Complex dielectric susceptibility at **c** O *K* edge and **d** Cu *L*_3,2_ edges.

One particular interest is to elucidate long-range order magnetic correlations of the doped holes in La_2-x_Ba_x_CuO_4_, which serves as a model system consisting of a few holes doped into a two-dimensional-*like* Cu-O plane separated by two La-O layers (Fig. 1a). While the electronic structure of the doped holes has been extensively studied, magnetic correlations of the doped holes remain elusive thus far.^2,3,6,7,9–15^ For the parent compound of La_2_CuO_4_ (or *x* = 0 in La_2-x_Ba_x_CuO_4_), soft X-ray absorption spectroscopy (XAS) measurements have shown that the lowest unoccupied state mostly consists of Cu3*d* hybridized with O2*p*, known as the upper Hubbard band (UHB), while the first excitation state (or the highest occupied state near the Fermi level) is mostly O2*p* in character, placing the cuprates as a charge transfer system in a Zaanen-Sawatzky-Allen picture (Fig. 1b)^16^. When Ba substitutes La (*x* > 0), the Cu-O layer is doped with holes and the electronic structure changes dramatically due to many-body interactions. Measurements with XAS have shown that the doped hole goes into O2*p* creating an unoccupied state^17^, namely a hole-doped peak (HDP), just above the Fermi level with a few eV below the UHB. As a function of hole doping, the HDP increases and is accompanied by spectral weight transfer between the UHB and the HDP, thus revealing the importance of many-body electronic correlations in the cuprates (Fig. 1c)^18–21^. Long-range magnetic order of the doped holes, on the other hand, is far from being understood.

Here, we experimentally observe strong ferromagnetism of the quasiparticle doped holes in the new low-dimensional-Au/*d-*La_1.8_Ba_0.2_CuO_4_/LaAlO_3_(001) heterostructures (or HOSG-QDs/*d-*LBCO/LAO, where *d* is the film thickness of LBCO) using newly developed spin- and site-selective resonant soft X-ray and magnetic scattering (RSXMS). The *d* is varied from 4, 8 to 12 unit-cells (uc) to show the roles played by Cu-O layers. Interestingly, ferromagnetism of the doped holes is stronger at O and increases for thicker *d*. The low-dimensional Au, which consists of highly oriented single-crystalline gold quantum dots (HOSG-QDs), is a Mott-*like* insulator with an optical band gap of ~1.5–2 eV, and is different from bulk, thin-film gold, or gold nanoparticles,^22,23^. The observation of ferromagnetism is further supported with zero-field XMCD at Cu *L*_3,2_ and O *K* edge.

**Fig. 2:**
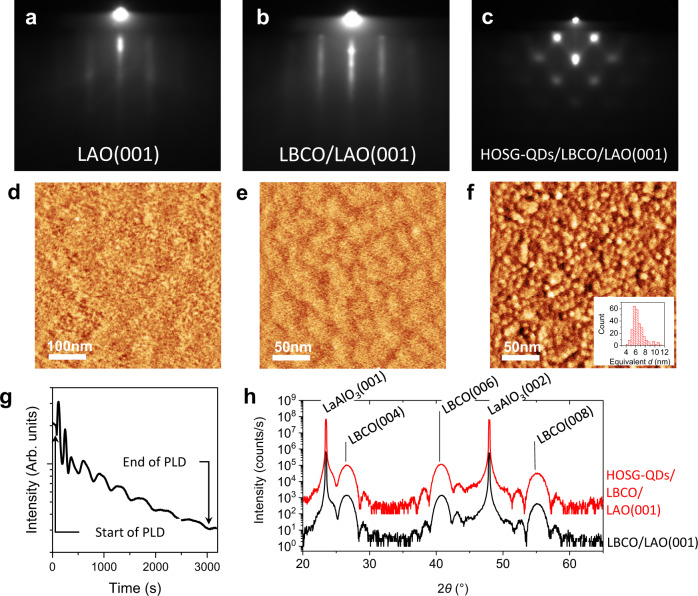
RHEED, XRD, and AFM images of HOSG-QDs/LBCO/LAO(001). **a**–**c** RHEED patterns of cleaned LAO(001), LBCO/LAO(001) and HOSG-QDs/LBCO/LAO(001). AFM corresponding surface morphologies are shown in **d**–**f**. **d** clean LAO(001) surface appears smooth with a surface roughness *R*_q_ of 0.14 nm. **e** the LBCO film surface appears smooth with *R*_q_ of 0.53 nm. **g** the HOSG-QDs have an average size of about 6.57 ± 1.28 nm (size-distribution shown in inset **f**). **g** RHEED intensity oscillations were observed during the PLD of LBCO. **h** *θ*−2*θ* scan of LBCO/LAO(001) with and without HOSG-QDs. Both samples show the presence of LBCO (I4/mmm phase).

Figure 2 shows the typical reflection high-energy electron diffraction (RHEED), high-resolution X-ray diffraction (HR-XRD), and atomic force microscopy (AFM) results obtained from the growth and characterization of HOSG-QDs/*d*-LBCO/LAO heterostructure with *d* = 4uc as a representative. Evidence from in-situ RHEED specular spot intensity oscillations (Fig. 2a), together with the in-situ RHEED pattern exhibiting the same (1 × 1) surface symmetry pattern before and after the growth of LBCO (Fig. 2b, c) on LAO(001), are indicative of two-dimensional (2D) “layer by layer” epitaxial growth occurring during in-situ pulsed laser deposition. Ex-situ XRD *θ*−2*θ* scans show strong diffraction patterns dominated only by the LBCO film and LAO substrate peaks. The LAO (001) and (002) substrate peaks are at 23.44° and 47.92° (Fig. 2e)^24^, while LBCO (004), (006), and (008) film peaks are at 26.59°, 40.80° and 55.16°, respectively. Results of the XRD and RHEED analyses reveal LBCO belonging to the I4/mmm phase^25–27^ (see Supplementary Table 1 for details). It is noted that the *c*-axis lattice parameter of the LBCO film is revealed to be about 13.32 Å, which is typical for overdoped cuprates^28–31^. Collectively, these results suggest that epitaxial growth of LBCO film onto LAO(001) substrate has been realized with the following relationship given by {LBCO(001)//LAO(001) and LBCO < 001 > //LAO < 001 > }. Kiessig fringes are also observed in the XRD *θ*−2*θ* scans, and from the analysis of LBCO(006) associated fringes, the epitaxial LBCO film thickness is extracted to be ~5.5 ± 0.5 nm, which is equivalent to 4uc of LBCO. At this thickness, the surface has a root mean square (rms) roughness of ~0.53 nm, which is comparable to the initial rms surface roughness of LAO (Fig. 2d, e). Subsequent pulsed laser deposition of Au onto this LBCO surface leads to a three-dimensional (3D) growth where highly oriented single-crystalline gold quantum dots (HOSG-QDs)^32^ are formed (Fig. 2f). The original streak RHEED pattern observed from the LBCO surface is replaced by the appearance of a spotty Au pattern with an epitaxial relationship given by {Au(110)//LBCO(001) and Au[0$\bar{1}$1]//LBCO[0$\bar{1}$0]}. Details of the RHEED pattern analyses are shown in Supplementary Fig. 1. The same growth processes and structural properties are achieved for thicker *d* = 8 and 12uc cuprate heterostructures. As a comparison, 4uc LBCO/LAO with the same lattice constants but without HOSG-QDs is also grown.

The complex dielectric susceptibility, *χ*(ω) = Re *χ*(ω) + Im *χ*(ω) of HOSG-QDs/4uc LBCO/LAO and 4uc LBCO/LAO is obtained at the O *K* edge (Fig. 1c) and Cu *L*_3,2_ edges (Fig. 1d) as a representative. XAS is analyzed in detail by computing χ(ω). It is proportional to ${{\mbox{Im}}}\chi (\omega )$, and can be related through absorption coefficient, $\mu \left(\omega \right)=\frac{4\pi }{\lambda }{{\mbox{Im}}}{N}(\omega )$ and $N(\omega )=\sqrt{1+\chi (\omega )}={n}(\omega )+{i\; K}(\omega )$, where *N*(*ω*), *n*(*ω*) and *K*(*ω*) are the complex refraction index, refractive index, and extinction coefficient respectively, as a function of photon energy ω. From *μ*(ω), we calculate *K*(ω) and perform a Kramers-Kronig Transformation (KKT) to get *n*(ω), then compute *χ*(ω). The XAS are measured in total electron yield mode and a broad energy range from 3.7 eV to 1500 eV covering O *K*, Ba *M*_4,5_, La *M*_4,5_, and Cu *L*_3,2_ edges to achieve stabilized KKT. The *K*(*ω*) shows that the chemical composition and stoichiometry for all cuprate heterostructures are all the same with *x* ~0.20 and no impurity is detected to the limit of the XAS (Supplementary Fig. 2)^17,33^. The *χ*(ω) shows that HOSG-QDs/*d*-LBCO/LAO (for *d* = 4, 8, and 12uc) and 4uc LBCO/LAO have similar electronic structures at Cu *L*_3,2_ edges but some differences at the O *K* edge. The XAS at the O *K* edge exhibits two pre-peaks: (i) the HDP at ~528.9 eV, which corresponds to a transition from O1*s* to the hole-doped peak in O2*p*, and (ii) the UHB at ~530.1 eV, which corresponds to a transition from O1*s* to Cu3*d* hybridized with O2*p* (Fig. 1b, c). Both pre-peaks are strongly coupled through many-body electronic correlation yielding spectral weight transfer. They are the center of discussion for decades because they contain critical information on the nature of magnetic and electronic correlations, particularly the charge carriers (doped holes or electrons), which are believed to determine the “magnetic glue” in the high-*T*_*c*_ superconductors. For the undoped cuprate (or parent compound), the XAS at O *K* edge shows only the UHB peak^17^. Upon hole doping, the hole-doped peak grows accompanied by spectral weight transfer between UHB and HDP^19,21^. Therefore, both pre-peaks contain strong doped hole (charge carrier) and magnetic characters. As a function of *d*, the overall HDP and UHB increases. By fitting the HDP and UHB using a Gaussian profile (Supplementary Fig. 3 and Supplementary Table 2) the ratio of HDP to UHB is ~0.60 ± 0.05 revealing the same total number of doped holes for all films. Such a ratio is in the range of overdoped cuprate films^34^. Note that the ratio of HDP to UHB for thin films is different than that of bulk samples due to the effects of the substrate and strain, which also lead to spectral weight transfer. Furthermore, the overall spectral weight at the O*K* edge of HOSG-QDs/*d*-LBCO/LAO is reduced compared to that of LBCO/LAO supporting the presence of HOSG-QDs at the top of LBCO (c.f. Fig. 1c and Supplementary Fig. 2a). The Cu *L*_3,2_ edges, on the other hand, show two main peaks, at ~931.0 eV and ~951.0 eV, which correspond to a transition from Cu2*p* into Cu3*d* where the hole has *j* = 1/2 or 3/2, respectively (Fig. 1b, d and Supplementary Fig. 2d). The hole-doped peak occurs at ~931.9 eV (Cu *L*_3_’), which is a transition from Cu2*p* to Cu3d*L*, where *L* is a ligand hole^35^.

In order to reveal long-range order of magnetic and electronic correlations of the doped holes in O (~528.9 eV of HDP) and in Cu (~530.1 eV of UHB and ~931.9 eV of Cu *L*_3_’) of the Cu-O-planes, we construct a spin- and site-selective RSXMS, which is a combination of magnetic scattering and valence spectroscopy (Fig. 1b). The RSXMS measurements are conducted in reflectivity mode using left and right circularly polarized photons (LCP and RCP, respectively) and by varying the angle of incidence, *θ*, resulting in a momentum transfer ***Q***_***HKL***_=(00 ***L***). The RSXMS is measured over an almost full range of *θ* from ~10° to ~88° corresponding to ***Q*** intervals (0,0,0.34) → (0,0,2.00) at Cu *L*_3_’ and (0,0,0.02) → (0,0,1.14) at HDP and UHB of O *K* edge and enabling the in-plane (at lower incident angles) and out-of-plane (at higher incident angles) magnetism to be probed. Ferromagnetism occurs when magnetic dichroism, which is defined as the difference between LCP and RCP, exists. To complement RSXMS, X-ray magnetic dichroism (XMCD) measurements at O *K* edge and Cu *L*_3,2_ edge are also performed. To further demonstrate the resonance effects, we also perform RSXMS at ~927.9 eV, which is an off (magnetic) resonance. It is worth highlighting that the scattering matrix element of RSXMS, ${M=\hat{{{{{{\boldsymbol{\varepsilon }}}}}}}}_{{CP},1}\times {\mathop{\chi }\limits^{\leftrightarrow}}_{{tot}}\left(\omega ,Q\right)\times {\hat{{{{{{\boldsymbol{\varepsilon }}}}}}}}_{{CP},2}$, and its intensity, ${I}_{{RSXMS}}\left(\omega ,Q\right) \sim {\left|M\right|}^{2}$, where ${\mathop{\chi }\limits^{\leftrightarrow}}_{{tot}}\left(\omega ,Q\right)$ and ${\hat{{{{{{\boldsymbol{\varepsilon }}}}}}}}_{{CP},1}$ (${\hat{{{{{{\boldsymbol{\varepsilon }}}}}}}}_{{RCP},2}$) are the tensor of susceptibility and incoming (outgoing) photon with circular polarization, respectively. Therefore, RSXMS can select specifically a momentum transfer, $Q=4\pi / \lambda {{\sin }}(\theta )$, and thus is highly sensitive to long-range order of spins (Supplementary Note 1).

**Fig. 3:**
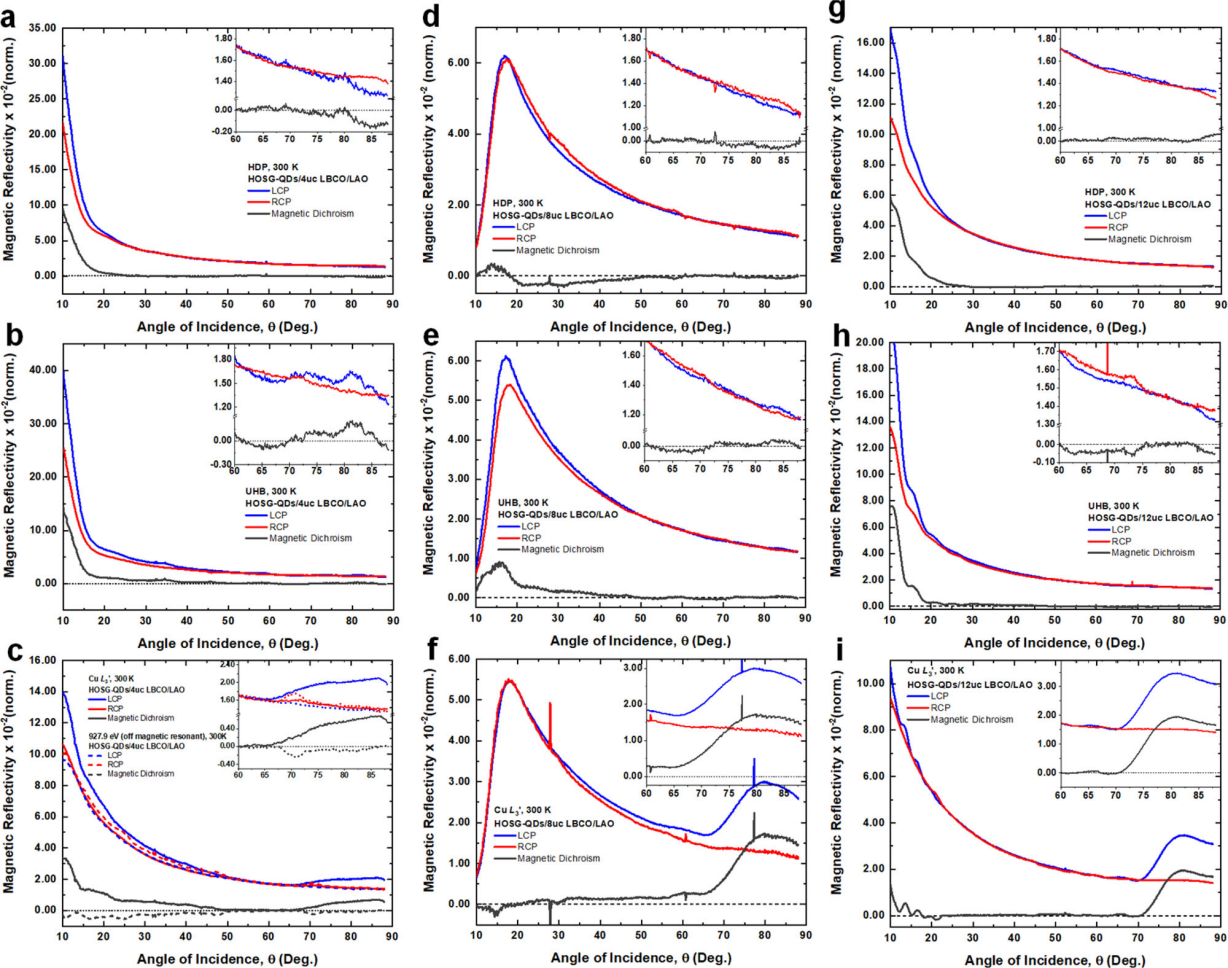
Room temperature resonant soft X-ray and magnetic scattering (RSXMS). RSXMS at room temperature: for HOSG-QDs/4uc LBCO/LAO at **a** HDP, **b** UHB, and **c** Cu *L*_3_’ and 927.9 eV (off-resonance); for HOSG-QDs/8uc LBCO/LAO at **d** HDP, **e** UHB, and **f** Cu *L*_3_’; and for HOSG-QDs/12uc LBCO/LAO at **g** HDP, **h** UHB, and **i** Cu *L*_3_’. Insets show the magnified data at higher incident angles.

A key observation is the presence of strong magnetic dichroism occurring at these resonance photon energies and even at a high temperature of 300 K for all cuprate heterostructures (Fig. 3). This directly reveals that the spin of doped holes in the O (HDP and UHB) and Cu (UHB and Cu *L*_3_’) are long-range order ferromagnetic (Fig. 1b). For HOSG-QDs/4uc LBCO/LAO, ferromagnetism is found to be anisotropic and stronger at low *θ* revealing that the in-Cu-O-plane (or *ab*-plane) is the magnetic easy-axis. For the in-plane spin-polarization, the strong magnetic dichroism at ~528.9 eV (~530.1 eV) shows that the spin of the doped hole (UHB) is coupled ferromagnetically between O2*p*–O2*p* (Cu3*d*–Cu3*d*) as seen in Fig. 3a (Fig. 3b). Furthermore, since the magnetic dichroism at both ~528.9 and ~530.1 eV occurs within the same direction, i.e., positive, this reveals the presence of ferromagnetic coupling between O2*p*‒O2*p*, Cu3*d*–O2*p*, and Cu3*d*–Cu3*d*. Such a strong ferromagnetism highlights the importance of the spin-triplet state of the doped holes between Cu3*d*‒O2*p* (Fig. 1a)^8^. For the out-of-*ab*-plane spin-polarization, magnetic dichroism is still present but relatively weaker compared to that of the in-plane spin-polarization. Based on the direction of magnetic dichroism, it is found that while the spin of the doped holes and UHB is coupled ferromagnetically (spin triplet state) in both Cu3*d*‒Cu3*d* and O2*p*–O2*p*, the spin of doped holes and UHB is coupled antiferromagnetically (spin singlet) in O2*p*‒Cu3*d*. This suggests that both spin triplet and singlet states are present for out-of-plane spin-polarization yielding ferrimagnetism. Such an interplay of spin-triplet and singlet states results in anisotropy between in- and out-of-plane ferromagnetism in the lightly hole-doped cuprate.

Another significant result is that we observe a new ferromagnetic-(002) Bragg peak of the doped holes at higher *θ*~80°, which is commensurate and broad due to a finite number of Cu-O layers (~4uc), at ~931.9 eV (Fig. 3c). Interestingly, the ferromagnetic-(002) Bragg peak presents strong dichroism, where it is stronger in LCP and weak in RCP. This demonstrates that for out-of-plane spin-polarization, the spin of the doped holes is coupled ferromagnetically along the *c*-axis (inter Cu-O layer) as illustrated in Fig. 1a. For comparison, neither in-plane nor out-of-plane dichroism is observed at off magnetic resonance of ~927.9 eV, revealing that the ferromagnetism is solely due to doped holes. As a function of increasing *d*, the intensity of ferromagnetic-(002) Bragg peak of the quasiparticle doped holes enhances, and the full width at half maximum of the hole-doped ferromagnetic-(002) Bragg peak decreases following the film thickness, i.e., when the thickness of the film increases, the Bragg peak becomes sharper (Fig. 3d, i). Upon heating, the intensity of the Bragg peak is reduced. Note that the individual sample rocking scan and detector scans are also performed (Supplementary Fig. 4). These observations fully confirmed that the Bragg peak at this higher *θ* is truly ferromagnetic-(002) Bragg peak arising from the entire film, i.e. a bulk property. Thus, ferromagnetism is a distinct and unique property in the phase diagram of cuprates and occurs as a broader phenomenology of the Cu-O plane.

**Fig. 4:**
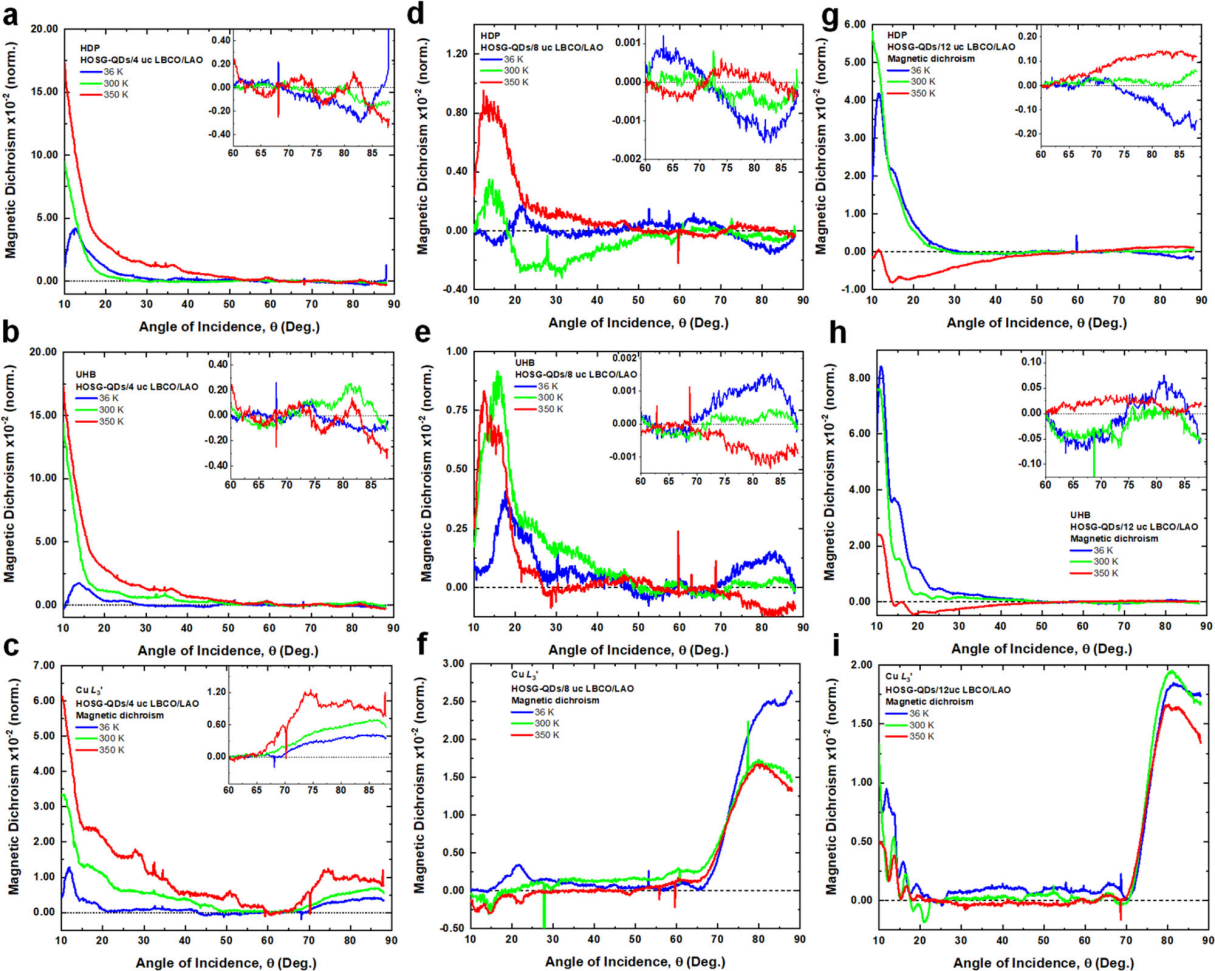
Temperature-dependent RSXMS. RSXMS measurements at *T* = 36 K, 300 K and 350 K revealing the magnetic dichroism: for HOSG-QDs/4uc LBCO/LAO at **a** HDP, **b** UHB, and **c** Cu *L*_3_’; for HOSG-QDs/8uc LBCO/LAO at **d** HDP, **e** UHB, and **f** Cu *L*_3_’; and for HOSG-QDs/12uc LBCO/LAO at **g** HDP, **h** UHB, and **i** Cu *L*_3_’. Insets show the magnified data at higher incident angles.

Figure 4 shows the temperature-dependent RSXMS at the same resonant photon energies for all cuprate heterostructures. For HOSG-QDs/4uc LBCO/LAO and using 300 K as a reference, the magnetic dichroism (both in-plane and out-of-plane) is significantly enhanced at higher temperature (350 K) but is notably suppressed at low temperature (36 K). This demonstrates that at higher temperatures (350 K), the spin-triplet state of the doped holes in Cu3*d*‒O2*p*‒Cu3*d* is dominating, yielding stronger ferromagnetism. Upon cooling to a lower temperature (36 K), while the spin-triplet state remains, the spin-singlet state of the doped holes in Cu3*d*‒O2*p* is enhanced yielding ferrimagnetism. As a function of *d* and temperature, the in-Cu-O-plane ferromagnetism of the doped holes decreases but remains significantly large (Fig. 4d, i). This reveals that the coupling between Cu-O layers along the *c*-axis plays an important role in determining the long-range order of the spin of the doped holes within the *ab*-plane (c.f. Fig. 1a).

**Fig. 5:**
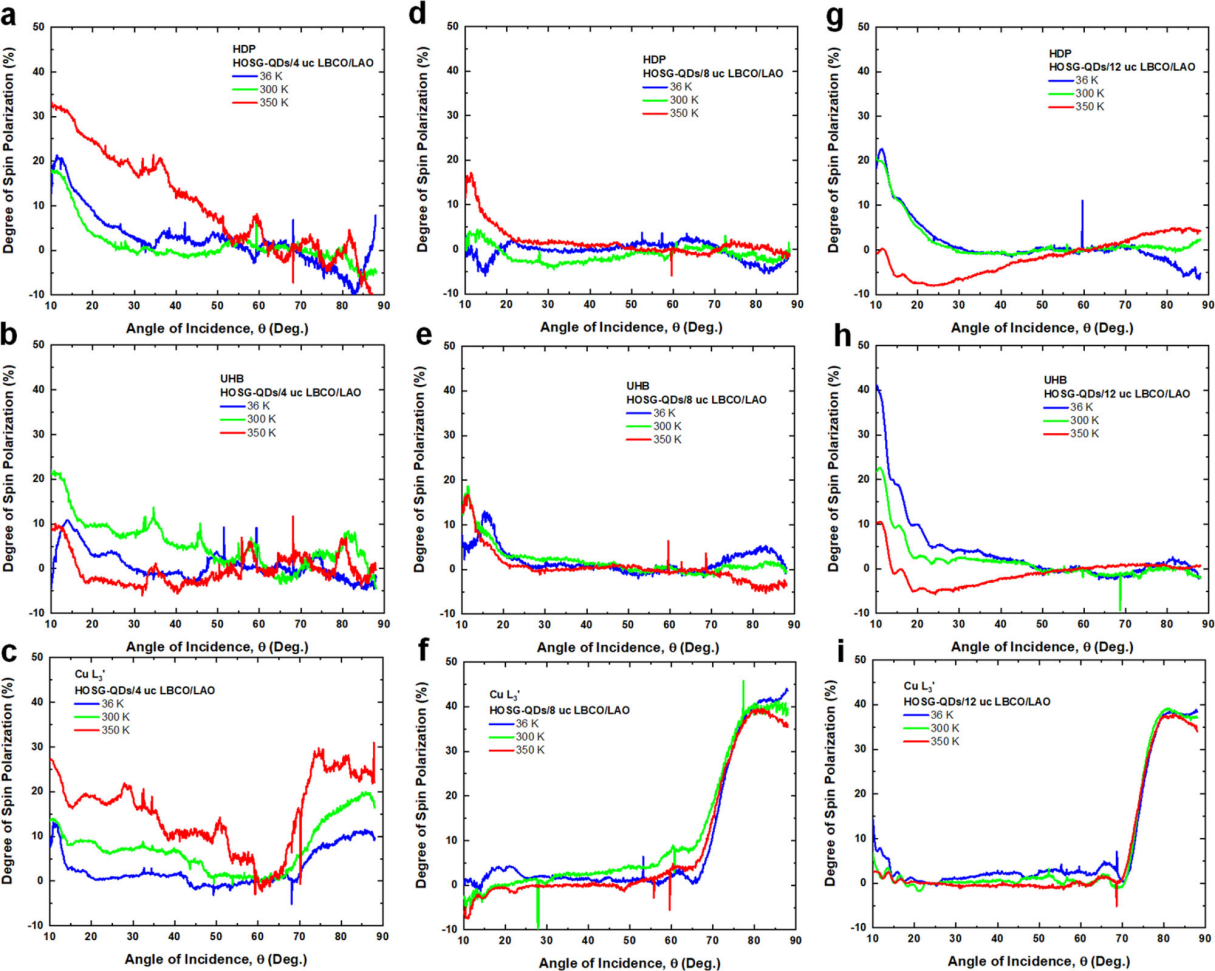
Degree of spin polarization as a function of temperature. Degree of spin-polarization at *T* = 36 K, 300 K, and 350 K: for HOSG-QDs/4uc LBCO/LAO at **a** HDP, **b** UHB, and **c** Cu *L*_3_’; for HOSG-QDs/8uc LBCO/LAO at **d** HDP, **e** UHB, and **f** Cu *L*_3_’; and for HOSG-QDs/12uc LBCO/LAO at **g** HDP, **h** UHB, and **i** Cu *L*_3_’.

Figure 5 shows the degree of spin-polarization (DSP) of the doped holes, (DSP ≡ (LCP-RCP)/(LCP+RCP)×100%), which determines the strength of ferromagnetism. We start with the *ab*-plane ferromagnetism of the doped holes. For HOSG-QDs/4uc LBCO/LAO, it is found that for in-plane spin-polarization the DSP is ~35%, which is the strongest at the O site (~528.9 eV) and it occurs remarkably even at 350 K (Fig. 5a–c). As a function of *d* and temperature, the DSP remains as high as ~40% at UHB and Cu *L*_3_’ (Fig. 5d, i). This reveals that while the ferromagnetism of the doped holes is in the in-Cu-O-plane, it is mostly at the O sites for the thinner films and at Cu sites for the thicker films. Next, we look at the ferromagnetic-(002) Bragg peak of doped holes. For HOSG-QDs/4uc LBCO/LAO the DSP of ferromagnetic-(002) Bragg peak is as strong as ~30% revealing that the out-of-plane ferromagnetic coupling along the *c*-axis is nearly as strong as the in-Cu-O-plane ferromagnetic coupling. As a function of increasing *d*, the DSP enhances and is also nearly temperature-dependent.

It is worth highlighting the implication of our experimental data to the ground state of the cuprates. One of the most essential yet unsolved issues in the cuprates is understanding how the magnetic correlations evolve in the vicinity of doped hole carriers, while considering the observed strong doping-dependent interplay between magnetism and superconductivity. Two main scenarios, which are hotly debated till today, are proposed to describe their mechanisms. On one hand, a local singlet character of the doped hole in the CuO_2_ planes with a net-zero spin moment, namely Zhang-Rice singlet (ZRS) is proposed^3^. On the other hand, a model of three-spin polaron is proposed where the doped hole in oxygen can promote local ferromagnetic fluctuations of Cu^2+^ spins surrounding the doped hole in an otherwise antiferromagnetic background.^2^ Our new experimental data reveal that the ground state of the cuprates is a strong mixture of spin triplet-and-singlet wavefunction resulting in a remarkably strong degree of spin-polarization and ferromagnetic correlations between Cu-spins near the doped holes (c.f. Fig. 1a). In order to further reveal that holes doped in the Cu-O planes of the LBCO/LAO heterostructures order ferromagnetically, we perform RSXMS on 4uc LBCO/LAO, which is the cuprate heterostructure without HOSG-QDs, and we find that both the in-Cu-O-plane and out-of-Cu-O-plane ferromagnetism of the doped holes are still present, albeit significantly reduced (Supplementary Fig. 5**)**. This shows that the ground state of the cuprates is a strong combination of spin triplet-and-singlet wavefunction yielding ferromagnetism. HOSG-QDs further enhance the spin-triplet state, and consequently increase the ferromagnetic long-range order strength of the quasiparticle doped holes in the Cu-O plane. Note that since HOSG-QDs are ferromagnetic Mott-*like* insulators with a spin-polarized Mott-*like* state around 1.5–2 eV^32^ which is located within the charge transfer gap in the cuprates^8,36^, spin and electronic correlations may, in turn, be enhanced between HOSG-QDs and LBCO. We further perform zero-field XMCD and find that the XMCD signals are indeed observed at both Cu *L*_3,2_ edges and at O *K* edge, particularly at HDP and UHB, therefore fully supporting the findings of ferromagnetism of the doped holes in the Cu-O planes (Supplementary Fig. 6 and Supplementary Note 1 in Supplementary Figure). Since both UHB and HDP show strong magnetic dichroism from both RSXMS and XMCD, this also reveals ferromagnetism of the Mottness. We find that RSXMS is highly sensitive in detecting the spin of the doped holes, and together with XMCD, innately forms a compelling technique to unravel long-range order of spin of charge carriers.

In conclusion, we experimentally observe ferromagnetism of the quasiparticle doped holes with a high degree of spin-polarization in the Cu-O planes of new low-dimensional Au/*d*-La_1.8_Ba_0.2_CuO_4_/LaAlO_3_(001) heterostructures using a unique RSXMS technique combined with XMCD. Ferromagnetism of the doped holes persistent in 4uc La_1.8_Ba_0.2_CuO_4_/LaAlO_3_(001) reveals that the ground state of Cu-O planes consists of a strong mixture of spin triplet- and singlet-states. The increase in ferromagnetism in HOSG-QDs/4uc La_1.8_Ba_0.2_CuO_4_/LaAlO_3_(001) suggests that the low-dimensional HOSG-QDs enhance the spin-triplet state through the spin and electronic correlation effects, and in turn strengthens the long-range order of spin of the doped holes. Furthermore, it is also further enhanced in thicker LBCO films for HOSG-QDs/*d*-La_1.8_Ba_0.2_CuO_4_/LaAlO_3_(001) heterostructures (with *d* up to 12uc) suggesting a strong ferromagnetic coupling between Cu-O layers along the *c*-axis. Our result also opens new possibilities to study the relationship between ferromagnetism and unconventional superconductivity and demonstrates a unique strategy in utilizing RSXMS to reveal spin, charge, and orbital degrees of freedom in strongly correlated electron systems.

## Methods

### Sample preparation

Highly oriented single-crystal gold quantum dots (HOSG-QDs) and LBCO films are prepared on 1 cm × 1 cm LAO(001) substrates by a unique ultrahigh vacuum molecular beam epitaxy pulsed laser deposition (UHV-MBE PLD system) equipped with a solid-state ablation Nd:YAG laser (laser output wavelength 266 nm) and growth monitoring using in-situ reflection high-energy electron diffraction (in-situ RHEED). Each LAO(001) substrate is rinsed in isopropanol and blown-dried in the air before being loaded into the UHV MBE PLD system with a base pressure of 5 × 10^−9^ Torr. The substrate is first outgassed in a vacuum at 310 °C for 45 mins before being annealed at 750 °C for 60 mins under 50 mTorr O_2_ to obtain a clean LAO(001) surface as verified using in-situ RHEED (see Supplementary Fig. 1). The LBCO film is then pulsed laser deposited onto LAO(001) at 750 °C under 50 mTorr O_2_. The sample is subsequently annealed at 750 °C under 300 Torr O_2_ for another 60 mins. Prior to Au deposition, the LBCO/LAO(001) sample is first reduced to the growth temperature of 450 °C. Au is then pulse-laser deposited at the growth temperature under 10 mTorr O_2_. The laser energy is fixed at about 3.25 J cm^−2^ for all depositions. The sample is then cooled down to room temperature under 10 mTorr O_2_ at 25 °C min^−1^, while all other temperature ramps are fixed at 50 °C min^−1^. Prior to each deposition stage, the LBCO and Au targets are pre-ablated at 10 Hz with 2000 pulses. Transport measurement suggests that LBCO/LAO films with thicknesses of up to 12uc are in the regime of insulator-metal transition as a function of temperature and thickness^30,31^.

### Resonant soft X-ray magnetic scattering, soft X-ray absorption spectroscopy, and X-ray magnetic circular dichroism

Resonant soft X-ray and magnetic scattering (RSXMS), soft XAS, and X-ray magnetic circular dichroism (XMCD) measurements are carried out in an ultrahigh vacuum (UHV) five-circle geometry of a magnetic scattering chamber with based pressure of 10^−9^ mbar at the soft X-ray-ultraviolet (SUV) beamline of the Singapore Synchrotron Light Source^33^. For RSXMS, the scattered photon is collected using a photodiode to get the total fluorescence yield (TFY) from the sample. X-ray photons with selectively left- and right-circular polarizations (LCP and RCP) are used with a degree of circular polarization of ~80% and the measurements are done at reflectivity mode in which the angle of incidence of the sample (the angle of the detector) is scanned in almost a full range from 10° to 88° (20° to 176°) as shown schematically in Fig. 1b. The temperature of the sample stage of the UHV magnetic scattering chamber can be varied and controlled in a fine step from 35 K to 400 K via control of the sample holder temperature with a temperature gauge on the sample. The SUV beamline covers the energy range from 3.5 to 1500 eV. The XAS and XMCD measurements are conducted using linearly- and circularly-polarized X-ray photons, respectively. The X-ray photon is then absorbed by the sample and produces an electric current, which is counted by a current meter to get the total electron yield of the sample. All the spectra are normalized to the incident X-ray photon intensity. The normalization procedure of RSXMS is as follows. Firstly, at off-resonance (~927.9 eV), where magnetic dichroism is absent or negligible, the scattering intensity is initially set as the general background for all RSXMS. Secondly, the outgoing photon is divided by the incoming photon (detected as *I*_o_). We find that at the range of *θ* ~50°−60°, which are quite far from the ferromagnetic-(002) Bragg peak at the high *θ* and magnetic dichroism at low *θ*, the RSXMS of LCP and RCP at on- and off-resonance are naturally lined up and thus we set this range of *θ* as the reference for the RSXMS. This background is then confirmed with XMCD, where there is no dichroism beyond resonance edges. We find that this approach of normalization is qualitatively solid for this case.

## Supplementary information

Supplementary Information

## Data Availability

All data that support the findings of this study are available from the corresponding author upon reasonable request.
