# Supplementary material for: Anomalous Ferromagnetism of quasiparticle doped holes in cuprate heterostructures revealed using resonant soft X-ray magnetic scattering
Source: Nat Commun. 2022 Aug 8;13:4639. doi: 10.1038/s41467-022-31885-1 (PMC9360448; doi:10.1038/s41467-022-31885-1)
Supplement: Supplementary file 1 — Supplementary Information [file 41467_2022_31885_MOESM1_ESM.pdf]

Supplementary Information

Anomalous Ferromagnetism of quasiparticle doped holes in cuprate heterostructures revealed using resonant soft X-ray magnetic scattering

A. Rusydi *et. al.*

Supplementary Notes

Supplementary Note 1. Scattering matrix element of RSXMS and XMCD

We find that dichroism in RSXMS is much stronger than that of XMCD and this is expected because the scattering matrix element between these two experiments is different. This can be qualitatively explained as follows. Let's have a material with the complex (dielectric and magnetic) susceptibility can be written as

$$\chi(\omega,\vec{\mathbf{Q}}) \equiv Re\,\chi(\omega,\vec{\mathbf{Q}}) + Im\,\chi(\omega,\vec{\mathbf{Q}}), \quad (1)$$

where ω is the (photon) energy and Q is the momentum transfer.

In particular, for magnetic dichroism, we consider complex susceptibility of spin (or magnetic part). Therefore, at Cu$L_{3,2}$, $\chi(\omega,\vec{\mathbf{Q}})$ can be separated into two parts:

$$\chi_{Cu}(\omega,\vec{\mathbf{Q}}) = \chi_{Cu,NM}(\omega,\vec{\mathbf{Q}}) + \chi_{DH,\uparrow}(\omega,\vec{\mathbf{Q}}) + \chi_{DH,\downarrow}(\omega,\vec{\mathbf{Q}}), \quad (2)$$

where $\chi_{Cu,NM}(\omega,\vec{\mathbf{Q}})$ is the complex susceptibility of non-magnetic Cu, while $\chi_{DH,\uparrow}(\omega,\vec{\mathbf{Q}})$ and $\chi_{DH,\downarrow}(\omega,\vec{\mathbf{Q}})$ are complex susceptibility of spin-up and spin-down of the doped hole in the Cu, respectively.

For the RSXMS, where $\vec{\mathbf{Q}} \neq \mathbf{0}$, scattering matrix element is different than that of XMCD. The scattering intensity of RSXMS can be written as

$$I_{RSXMS} = \left|\hat{\boldsymbol{\varepsilon}}_{LCP,1} \times \overleftrightarrow{\chi}_{tot}(\omega,\vec{\mathbf{Q}}) \times \hat{\boldsymbol{\varepsilon}}_{LCP,2}\right|^2 - \left|\hat{\boldsymbol{\varepsilon}}_{RCP,1} \times \overleftrightarrow{\chi}_{tot}(\omega,\vec{\mathbf{Q}}) \times \hat{\boldsymbol{\varepsilon}}_{RCP,2}\right|^2, \quad (3)$$

where $\hat{\boldsymbol{\varepsilon}}_{LCP,1}$ ($\hat{\boldsymbol{\varepsilon}}_{RCP,1}$) and $\hat{\boldsymbol{\varepsilon}}_{LCP,2}$ (($\hat{\boldsymbol{\varepsilon}}_{RCP,2}$) are the incoming photon with left circular polarized light (the incoming photon with right circular polarized light) and the out-going photon with left circular polarized light (the out-going photon with right circular polarized light), respectively.

We can estimate the enhancement of I_{RSXMS} at on magnetic-resonance Cu L_3' (~931.9 eV) compared to that at off magnetic-resonance (927.9 eV). From Eq. (3) and Fig.1d,

$$I_{RSXMS}/I_{off} \sim \left|\chi_{tot}\left(931.9eV, \overrightarrow{\boldsymbol{Q(HKL)}} = (\mathbf{002})\right)\right|^2 / \left|\chi_{tot}\left(927.9eV, \overrightarrow{\boldsymbol{Q(HKL)}} = (\mathbf{002})\right)\right|^2 = |(0.000547 - i\ 0.001563)|^2 / |(0.000260 - i\ 0.00100)|^2 \sim 2.5.$$

For the XMCD, where $\vec{\mathbf{Q}} \rightarrow 0$ and it is only considered for $Im\ \chi(\omega, \vec{\mathbf{Q}})$, the strength of XMCD can be written as

$$I_{XMCD}/I_{XAS} \sim \frac{\hat{\boldsymbol{\varepsilon}}_{LCP} \cdot Im\ \chi(\omega, 0) - \hat{\boldsymbol{\varepsilon}}_{RCP} \cdot Im\ \chi(\omega, 0)}{\hat{\boldsymbol{\varepsilon}}_{LCP} \cdot Im\ \chi(\omega, 0) + \hat{\boldsymbol{\varepsilon}}_{RCP} \cdot Im\ \chi(\omega, 0)}$$

$$\sim \frac{Im\ \chi_{DH,\uparrow}(\omega, 0) - Im\ \chi_{DH,\downarrow}(\omega, 0)}{2Im\ \chi_{Cu}(\omega, 0) + Im\ \chi_{DH,\uparrow}(\omega, 0) + Im\ \chi_{DH,\downarrow}(\omega, 0)} \tag{4}$$

Using Eq. 4, we estimate the I_{XMCD}/I_{XAS} for the doped holes (not the *intrinsic* holes from Cu^{2+}). For $La_{2-x}Ba_xCuO_4$ (x ~ 0.2), for a simplify case where the ratio of the total number of doped holes and the total number of intrinsic holes in Cu^{2+} per uc is 0.2/2 = 0.1 and the spins of the doped hole are all assumed lined up in one direction, then $\chi_{DH}(\omega, 0)$ can be estimated as $\chi_{DH,\uparrow}(931.9eV, 0) = 0.1 \times Im\ \chi_{Cu}(931.9eV, 0) = 0.1 \times 0.001563 \sim 1.563 \times 10^{-4}$ and $\chi_{DH,\downarrow}(\omega, 0) = 0$. This leads to I_{XMCD}/I_{XAS} ~ 0.05, which also sets the upper limit of the strength of XMCD at the Cu $L_{3,2}$ edges. Our XMCD at Cu L_3' (~931.9eV) (**Extended Data Fig. 6b**) show that the ratio of experimental data I_{XMCD}/I_{XAS} ~ 0.04, which is surprisingly close to our

theoretical estimation and further supports that the doped hole is highly spin polarized. Next, by comparing RSXMS and XMCD ($(I_{RSXMS}/I_{off})/(I_{XMCD}/I_{XAS})$), one would expect the enhancement as big as 2.5/0.05 ~ 50 times for RSXMS compared to that of XMCD. Note that at the O *K* edge, it is more complicated because of the many-body interaction yielding spectral weight transfer between HDP and UHB.

Supplementary Figures

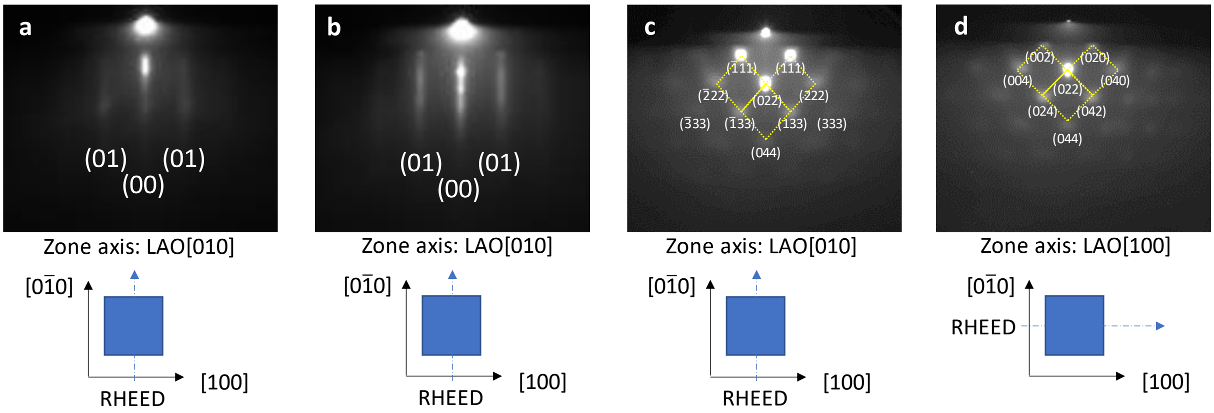

Supplementary Fig. 1. RHEED patterns. RHEED patterns obtained **a,** after annealing LAO(001) at 750°C, **b,** after depositing LBCO film at 750°C and **c-d**, after depositing Au onto LBCO/LAO(001) at 450°C. The streak pattern of LAO(001) remains largely unchanged after LBCO deposition showing a 2-D growth of LBCO where LBCO(001)//LAO(001) and LBCO<001>//LAO<001>. The spotted RHEED pattern of Au shown in **c-d** reveals a highly-oriented Au QDs which are single crystalline such that Au(011)//LAO(001) and Au[100]//LAO[100] and Au[0$\bar{1}$1]//LAO[0$\bar{1}$0].

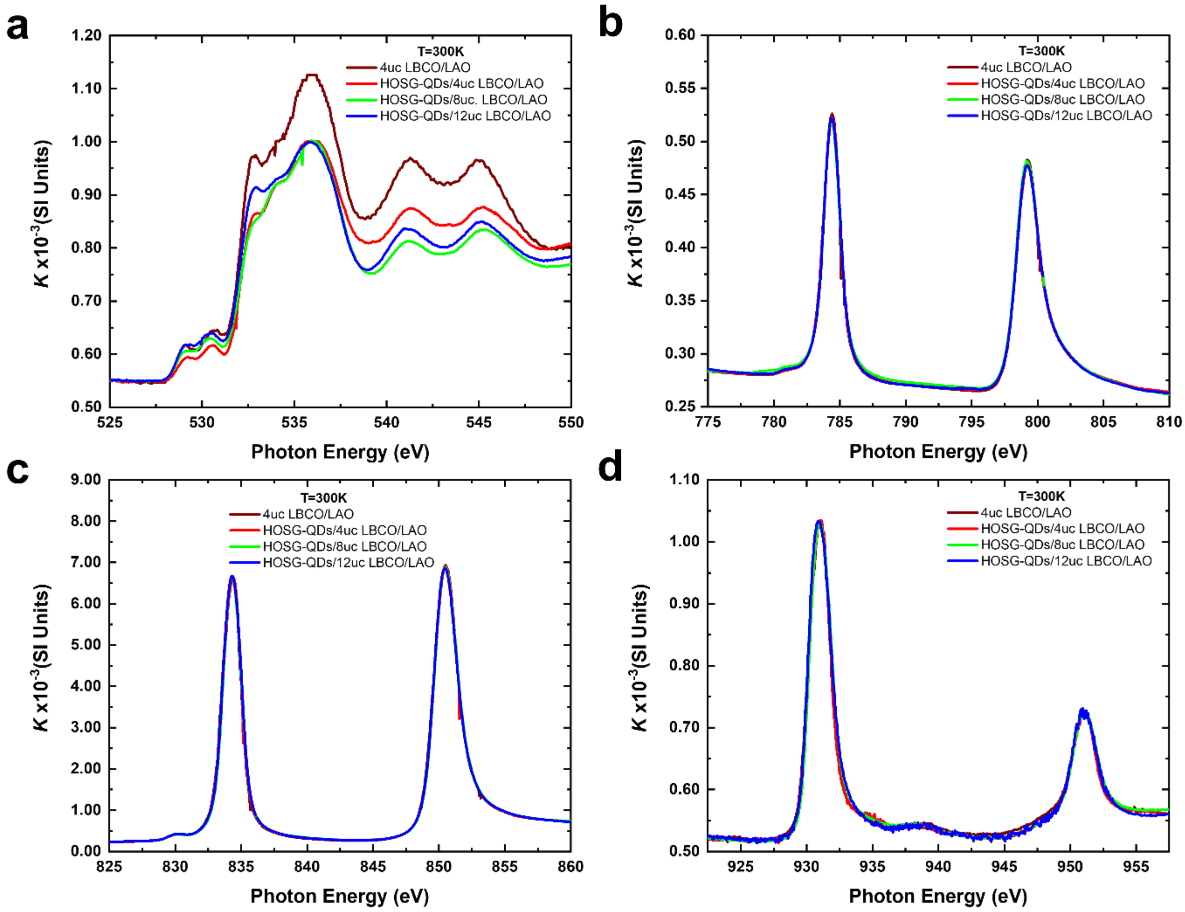

Supplementary Fig. 2. Extinction coefficient, K **of HOSG-QDs/4uc LBCO/LAO, HOSG-QDs/8uc LBCO/LAO, HOSG-QDs/12uc LBCO/LAO and 4uc LBCO/LAO. a,** O K, **b,** Ba $M_{4,5}$ **c,** La $M_{4,5}$, and **d,** Cu $L_{3,2}$ edges. The XAS measurements are done at 300 K.

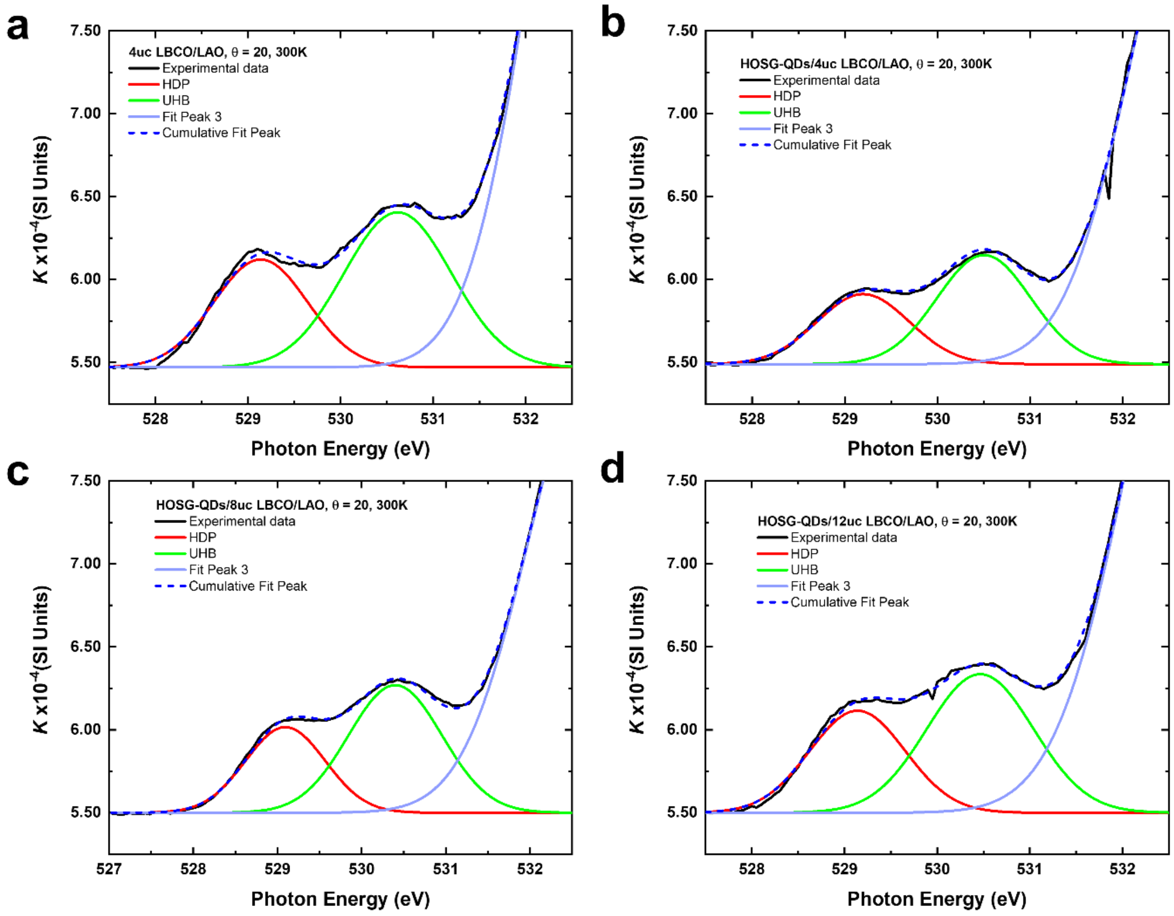

Supplementary Fig. 3. The HDP and UHB at O *K* edge and their fitting using gaussian profiles. a, 4uc LBCO/LAO and **b,** HOSG-QDs/4uc LBCO/LAO, **c,** HOSG-QDs/8uc LBCO/LAO and **d,** HOSG-QDs/12uc LBCO/LAO.

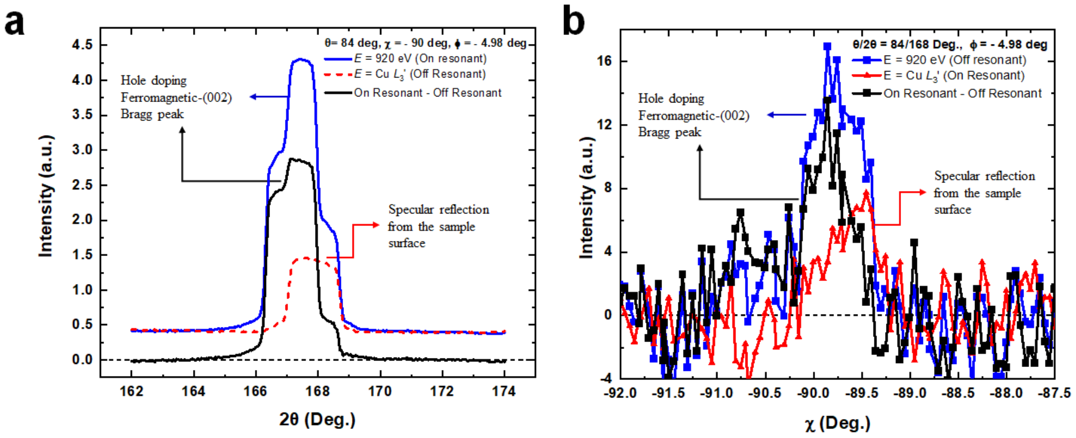

Supplementary Fig. 4. The sample and detector rocking scans taken at Cu L_3' eV (on resonance) and 920 eV (off-resonance). The experimental geometry is shown in Fig. 1b. (a) 2θ detector rocking scan around at $\theta = 84°$. At resonance edge, by setting up sample angle $\theta = 84°$ the hole doped ferromagnetic-(002) Bragg peak are clearly seen at detector angle 2θ ~167.5°. As comparison, at off resonance, Bragg peak is absent but instead a weak reflectivity due to specular reflection from the sample surface is observed. The plateau in the detector rocking scans is due to a wide opening angle of the photo-diode detector, however this does not affect $\theta / 2\theta$ scans. (b) The sample χ rocking scan at detector angle 2θ = 168°. The ferromagnetic-(002)-Bragg peak of the doped holes is clearly observed at on-resonance, while specular reflection is seen at off-resonance and they are well separated.

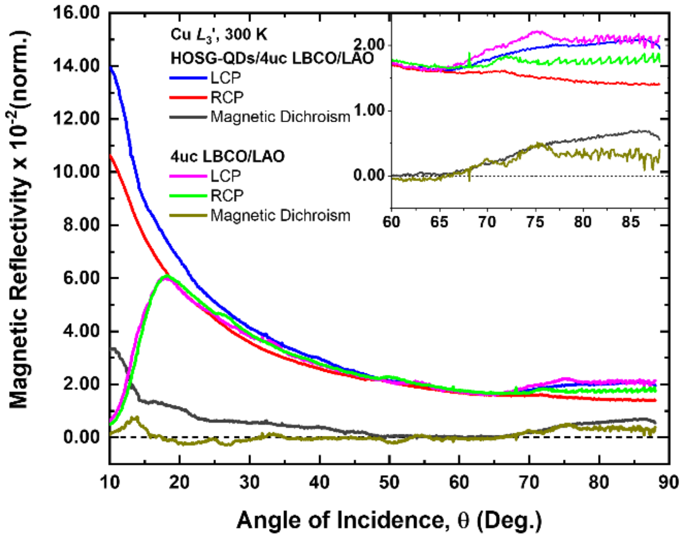

Supplementary Fig. 5. Comparison of room temperature RSXMS between HOSG-QDs/4uc LBCO/LAO and 4uc LBCO/LAO. Magnetic reflectivity for LCP and RCP incident beam and the resulting magnetic dichroism at room temperature are shown at Cu L_3' for both the samples. Inset shows the magnified data at higher incident angles.

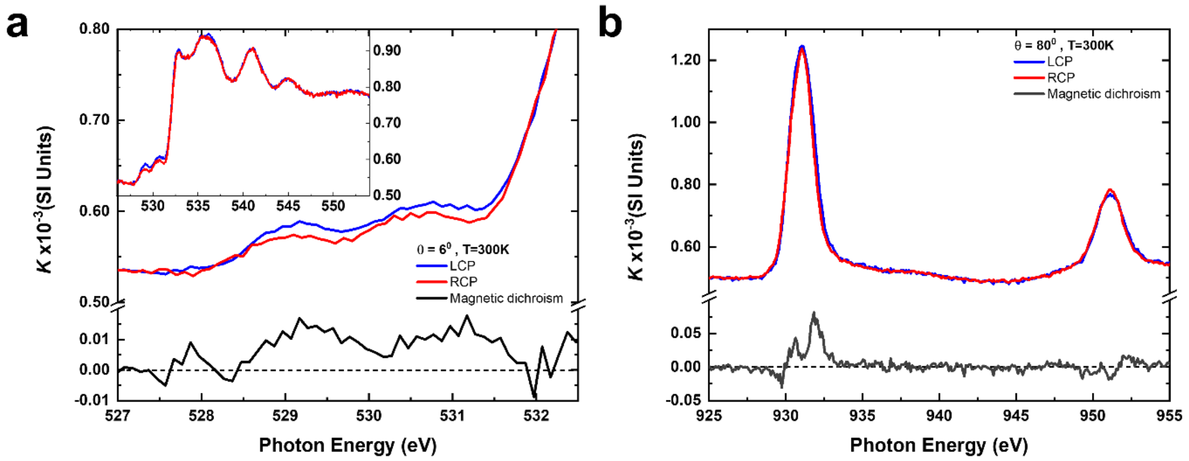

Supplementary Fig. 6. X-ray Magnetic Circular Dichroism (XMCD) for HOSG-QDs/4uc LBCO/LAO. Room temperature XMCD measurements near **a,** HDP and UHB at low incident angle (θ = 6º); and **b,** Cu L_3 edge at high incident angle (θ = 80º), revealing the magnetic dichroism observed. We select the experimental geometry where the RSXMS shows the strongest dichroism, i.e., at $\theta = 80°$ for XMCD at Cu $L_{3,2}$ edges where the ferromagnetic-(002) Bragg peak occurs, and $\theta = 6°$ for XMCD at O K edge where the magnetic fringes occur. Inset in **a** shows the full range of the O K-edge.

Supplementary Tables

Supplementary Table 1. XRD peak parameters. XRD peak parameters for HOSG-QDs/LBCO/LAO and LBCO/LAO samples, showing LAO(100) and (200) peaks, and LBCO(004), (006) and (008) peaks.

a

**LAO Peaks**	2θ	**d-spacing (Å) (λ = 1.5398 Å)**	**c-axis parameter (Å)**
(001)	23.44	3.790	3.79
(002)	47.92	1.896	3.79
		**Average**	3.79

b

**LBCO Peaks**	2θ	**d-spacing (Å) (λ = 1.5398 Å)**	**c-axis parameter (Å)**
(004)	26.58	3.35	13.39
(006)	40.80	2.21	13.25
(008)	55.16	1.66	13.30
		**Average**	13.32

Supplementary Table 2. Gaussian fit parameters used for 4uc LBCO/LAO, HOSG-QDs/4uc LBCO/LAO, HOSG-QDs /8uc LBCO/LAO and HOSG-QDs /12uc LBCO/LAO as shown in **Supplementary Fig. 3**.

**Sample**	**4uc LBCO/LAO**		**HOSG-QDs /4uc LBCO/LAO**		**HOSG-QDs /8uc LBCO/LAO**		**HOSG-QDs /12uc LBCO/LAO**	
**Peak**	**HDP**	**UHB**	**HDP**	**UHB**	**HDP**	**UHB**	**HDP**	**UHB**
x_c (Peak centre), eV	529.13	530.62	529.20	530.50	529.09	530.40	529.14	530.46
A (Area)	8.259×10^{-5}	1.364×10^{-4}	5.274×10^{-5}	8.347×10^{-5}	$6.125 x 10^{-5}$	1.071×10^{-4}	7.949×10^{-5}	1.204×10^{-4}
**FWHM,** eV	1.192	1.370	1.174	1.192	1.115	1.308	1.215	1.354

Supplementary Table 3. The Gaussian fit parameters of XMCD measurement on HOSG-QDs/4uc LBCO/LAO at Cu $L_{3,2}$ edges as shown in **Supplementary Fig. 6b**.

**Photon Polarization**	**LCP**		**RCP**	
**Peak**	**Cu L_3**	**Cu L_2**	**Cu L_3**	**Cu L_2**
x_c (Peak centre), eV	932.90	952.99	932.95	953.05
A (Area)	1.360×10^{-3}	5.440×10^{-4}	1.460×10^{-3}	4.990×10^{-4}
**FWHM,** eV	1.81	2.01	1.87	2.08
